# Supplementary material for: Temporary childbirth migration and maternal health care in India
Source: PLoS One. 2024 Feb 8;19(2):e0292802. doi: 10.1371/journal.pone.0292802 (PMC10852266; doi:10.1371/journal.pone.0292802)
Supplement: S1 Table — (DOCX) [file pone.0292802.s001.docx]

**SUPPLEMENTAL TABLE 1 (Appendix 1)**

*Table S1. Odds of temporary childbirth migration by sociodemographic characteristics among women 6-12 months postpartum, Bihar and Madhya Pradesh combined, 2019*

|  | Anytime during the perinatal period | During pregnancy | For delivery | Postpartum |
| --- | --- | --- | --- | --- |
|  | N=1,828 | N=1,289 | N=1,832 | N=1,289 |
|  | OR  (95% CI) | OR  (95% CI) | OR  (95% CI) | OR  (95% CI) |
| Age group (compared to <20) |  |  |  |  |
| 20-24 | 0.84 | 0.81 | 0.79 | 0.87 |
|  | (0.59 - 1.18) | (0.57 - 1.13) | (0.55 - 1.12) | (0.61 - 1.23) |
| 25-39 | 0.58** | 0.62* | 0.56** | 0.59** |
|  | (0.40 - 0.84) | (0.43 - 0.91) | (0.37 - 0.83) | (0.40 - 0.87) |
| 30+ | 0.36*** | 0.32*** | 0.34*** | 0.38*** |
|  | (0.23 - 0.56) | (0.20 - 0.49) | (0.21 - 0.56) | (0.24 - 0.61) |
| Women’s education group (compared to illiterate/no formal education) |  |  |  |  |
| Primary (1-5) | 1.16 | 1.27* | 1.16 | 1.15 |
|  | (0.92 - 1.47) | (1.00 - 1.60) | (0.89 - 1.50) | (0.90 - 1.48) |
| Some secondary (6-8) | 1.37** | 1.35* | 1.17 | 1.42** |
|  | (1.08 - 1.74) | (1.06 - 1.72) | (0.90 - 1.53) | (1.10 - 1.82) |
| Secondary (9-12) | 1.27* | 1.32* | 1.19 | 1.25 |
|  | (1.00 - 1.60) | (1.04 - 1.67) | (0.92 - 1.54) | (0.98 - 1.61) |
| More than secondary | 1.72* | 1.67* | 1.86** | 2.06** |
|  | (1.11 - 2.67) | (1.08 - 2.60) | (1.17 - 2.95) | (1.32 - 3.22) |
| Husband’s occupation (compared to daily non-agricultural |  |  |  |  |
| Agriculture | 1.20 | 1.24* | 1.07 | 0.93 |
|  | (0.98 - 1.48) | (1.01 - 1.53) | (0.85 - 1.34) | (0.75 - 1.16) |
| Salaried | 1.15 | 1.02 | 0.90 | 1.12 |
|  | (0.92 - 1.44) | (0.81 - 1.27) | (0.70 - 1.15) | (0.89 - 1.41) |
| Other | 1.10 | 1.21 | 0.72 | 0.71 |
|  | (0.71 - 1.71) | (0.78 - 1.88) | (0.43 - 1.21) | (0.44 - 1.16) |
| None | 1.92* | 1.27 | 0.84 | 1.20 |
|  | (1.02 - 3.64) | (0.67 - 2.40) | (0.41 - 1.70) | (0.62 - 2.30) |
| Woman works outside the home | 0.96 | 1.05 | 1.04 | 0.90 |
|  | (0.77 - 1.19) | (0.85 - 1.31) | (0.82 - 1.32) | (0.72 - 1.14) |
| Hindu (compared to other) | 0.65** | 0.77 | 0.79 | 0.86 |
|  | (0.49 - 0.88) | (0.57 - 1.04) | (0.57 - 1.08) | (0.63 - 1.17) |
| First birth (compared to second or higher) | 1.31** | 1.49*** | 1.33** | 1.22 |
|  | (1.08 - 1.59) | (1.23 - 1.82) | (1.08 - 1.65) | (1.00 - 1.50) |
| Wealth quintile (compared to poorest) |  |  |  |  |
| Poor | 1.04 | 1.00 | 1.05 | 1.03 |
|  | (0.81 - 1.34) | (0.78 - 1.28) | (0.79 - 1.39) | (0.79 - 1.35) |
| Middle | 1.27 | 1.06 | 1.11 | 1.03 |
|  | (0.99 - 1.63) | (0.82 - 1.36) | (0.83 - 1.47) | (0.78 - 1.36) |
| Wealthy | 1.51** | 1.24 | 1.22 | 1.24 |
|  | (1.16 - 1.95) | (0.96 - 1.61) | (0.91 - 1.63) | (0.94 - 1.63) |
| Wealthiest | 1.44* | 1.10 | 1.38* | 1.42* |
|  | (1.09 - 1.89) | (0.83 - 1.45) | (1.02 - 1.87) | (1.06 - 1.90) |

*Notes:*

*Temporary childbirth migration is defined as being away from the marital home for at least one month during the perinatal period (or specified component of the perinatal period).*

*Mixed methods logistic regression model accommodating clustering at the district and village levels and to provide a population-averaged estimate.*

*P-values for categorical comparison of odds of index category with reference category: *** p<0.01, ** p<0.05, * p<0.1*

*CI: Confidence intervals; OR: Odds ratios*
