# Supplementary material for: Temporary childbirth migration and maternal health care in India
Source: PLoS One. 2024 Feb 8;19(2):e0292802. doi: 10.1371/journal.pone.0292802 (PMC10852266; doi:10.1371/journal.pone.0292802)
Supplement: S2 Table — (DOCX) [file pone.0292802.s002.docx]

**SUPPLEMENTAL TABLE 2 (Appendix 2)**

*Table S2: Relationship between temporary childbirth migration during the perinatal period and facility delivery among women 6-12 months postpartum, Bihar and Madhya Pradesh, 2019*

|  | **Facility Delivery** | |
| --- | --- | --- |
|  | **Bihar (N=1,832)** | **MP (N=1,289)** |
|  | **OR (95% CI)** | **OR (95% CI)** |
| Temporary Childbirth Migration |  |  |
| Months at Natal Home, 3rd Tri |  |  |
| Natal Home at Childbirth | 1.37 (0.98 - 1.89) | 0.01 (0.00 - 17.20) |
| Natal Home Postpartum |  |  |
| Age Group |  |  |
| <20 |  |  |
| 20-24 |  |  |
| 25-29 |  |  |
| 30+ |  |  |
| Educational Attainment |  |  |
| None | REF | REF |
| Primary (1-5) | 1.30 (0.91 - 1.86) | 5.77 (0.80 - 41.8) |
| Some secondary (6-8) | 1.71** (1.15 - 2.54) | 10.6* (1.25 - 90.4) |
| Secondary (9-12) | 2.37*** (1.65 - 3.40) | 177** (6.35 - 4,920) |
| More than secondary | 3.04** (1.35 - 6.85) | 586* (1.08 - 316,590) |
| Husband Occupation |  |  |
| Daily non-agricultural |  |  |
| Agriculture |  |  |
| Salaried |  |  |
| Other |  |  |
| None |  |  |
| Respondent Works Outside Home |  |  |
| Hindu vs. Other Caste | 1.63* (1.03 - 2.58) | 0.34 (0.0057 - 20.0) |
| First Birth vs. Higher | 2.10*** (1.51 - 2.93) | 23.9** (2.61 - 218) |
| Household Wealth Quintile |  |  |
| Lowest quintile | REF | REF |
| Lower-middle quintile | 1.22 (0.87 - 1.72) | 2.97 (0.50 - 17.5) |
| Middle quintile | 1.44 (0.99 - 2.10) | 21.1* (1.76 - 252) |
| Higher-middle quintile | 2.24*** (1.48 - 3.38) | 9.80 (0.98 - 97.7) |
| Highest quintile | 1.78* (1.11 - 2.84) | 13.2* (1.01 - 172) |
| District |  |  |
| 1 | REF | REF |
| 2 | 1.78* (1.14 - 2.79) | 0.11 (0.00 - 3.84) |
| 3 | 2.35*** (1.42 - 3.87) | 0.00** (0.00 - 0.028) |
| 4 | 1.99** (1.23 - 3.21) | 0.00** (0.00 - 0.03) |
| 5 | 2.55*** (1.54 - 4.20) | 0.00** (0.00 - 0.03) |
| 6 | 3.79*** (2.23 - 6.45) | 0.00** (0.00 - 0.02) |
| Random parameters |  |  |
| M1[village]^a^ | 1 (const) | 1 (const) |
| L [individual]^a^ | - | 7.82 (11.10) |
| **Temporary Childbirth Migration Variable** | **Natal home, for childbirth** | |
| Maternal Age Group |  |  |
| <20 | REF | REF |
| 20-24 | 0.81 (0.54 - 1.23) | 0.94 (0.44 - 2.01) |
| 25-29 | 0.62* (0.39 - 0.98) | 0.52 (0.22 - 1.24) |
| 30+ | 0.36*** (0.20 - 0.63) | 0.33* (0.12 - 0.94) |
| Maternal Educational Attainment |  |  |
| None |  |  |
| Primary (1-5) |  |  |
| Some secondary (6-8) |  |  |
| Secondary (9-12) |  |  |
| More than secondary |  |  |
| Husband Occupation |  |  |
| Daily non-agricultural |  |  |
| Agriculture |  |  |
| Salaried |  |  |
| Other |  |  |
| None |  |  |
| Respondent Works Outside Home | 0.54** (0.36 - 0.80) | 1.73** (1.18 - 2.53) |
| Hindu vs. Other Caste |  |  |
| First Birth vs. Higher | 1.40* (1.05 - 1.85) | 1.64* (1.07 - 2.50) |
| Household Wealth Quintile |  |  |
| Lowest quintile |  |  |
| Lower-middle quintile |  |  |
| Middle quintile |  |  |
| Higher-middle quintile |  |  |
| Highest quintile |  |  |
| District |  |  |
| 1 | REF | REF |
| 2 | 1.36 (0.90 - 2.06) | 1.36 (0.90 - 2.06) |
| 3 | 1.29 (0.85 - 1.95) | 1.29 (0.85 - 1.95) |
| 4 | 1.93** (1.30 - 2.87) | 1.93** (1.30 - 2.87) |
| 5 | 2.77*** (1.84 - 4.18) | 2.77*** (1.84 - 4.18) |
| 6 | 1.73** (1.16 - 2.58) | 1.73** (1.16 - 2.58) |
| Random parameters |  |  |
| M1[village]^a^ | -0.36 (0.13) | 0.14 (0.08) |
| L [individual]^a^ | - | 1 (const) |
| Random parameters |  |  |
| var(M1[village])^b^ | 0.70 (0.19) | 10.82 (8.92) |
| var(L[individual])^b^ | - | 0.49 (1.30) |

*Notes:*

*OR: Odds ratio; ^a^ β(SE), ^b^Var(SE)*

*Analysis represents joint modeling of 1) sociodemographic characteristics on temporary childbirth migration and 2) temporary childbirth migration on perinatal care receipt.*

*Mixed effects models were used to accommodate clustering at the village level due to sampling and to provide a population-averaged estimate. District effects are modeled as fixed-effects to explicitly account for time invariant district specific characteristics.*

*Where no selection was identified, this parameter was removed from the final model and is noted in the table above by (–) within the var (L) row.*
