# Supplementary material for: Temporary childbirth migration and maternal health care in India
Source: PLoS One. 2024 Feb 8;19(2):e0292802. doi: 10.1371/journal.pone.0292802 (PMC10852266; doi:10.1371/journal.pone.0292802)
Supplement: Attachment — Submitted filename: Temporary childbirth migration and maternal health care in India - Author Comments.pdf [file pone.0292802.s003.pdf]

Temporary childbirth migration and maternal health care in India – Comments for Authors

Overall, I think this paper is interesting and does paper does contribute to the literature by documenting an under investigated phenomena. The paper does contribute a documentation of an under investigated phenomena which potentially has some profound implications for policy. The analysis, although limited in scope does appear to be a reasonable approach. The discussion and conclusion do follow from the results. However, the paper does not extend much beyond documenting this phenomenon. Given that the main contribution of the paper is to document the phenomena, it may be worthwhile emphasising why this is important to document and what implications these results have, especially for health outcomes, which I believe are not covered sufficiently.

I can see that documenting this phenomenon is potentially important for policymakers and for future research. For researchers, not knowing that mothers migrate during pregnancy or soon thereafter is likely causing survey sampling issues because they migrate. Also, the authors make clear that many policies designed to support mothers during pregnancy are not designed to accommodate maternal migration, and therefore is important to document. Indeed, the authors say specialised healthcare workers say that there "is no way to track women who leave for their mother's village or identify women who come to their village because it was where their parents lived." In terms of future research, it is important to understand the health consequences on mothers and their children from migrating during pregnancy or postpartum. The analysis and discussion on health outcomes specifically is very limited, and I would have liked at least a discussion of likely impacts even if a formal analysis was not possible. Although, if associations between migration and health outcomes are possible to estimate they should be included. The discussion around health implications of this phenomena on mothers and babies is limited and I think this needs expanding, given that this is the key reason why it is important to document this phenomena.

I have several suggestions and comments which require attention prior to publication, none of which are particularly substantive but do require revisions to the manuscript. The comments are presented chronologically.

<u>Section 1:</u> Is there any evidence of childbirth migration in developed countries? If so, do motivations differ? This may not be relevant but add if there is literature on this.

<u>Line 147 to line 152:</u> I think these beliefs of ritual pollution warrants a little more discussion. Could it be that this is a driver of migration? If mother "should only be touched by close female relatives including grandmothers" then this could almost fully explain reasons for returning to natal home. It would be useful to provide a statistic of how widespread of a belief this is, and a very small amount

more explanation of the belief and its repercussions. It is interesting that mothers do not mention it as a motivator for moving. I would have also expected this to be included specifically in the quantitative component of the analysis, not simply “for cultural reason”. It could be that this type of belief is driving mothers to their natal homes because they receive no care in their family home as a result of these beliefs. If these beliefs mean they receive no care in their family home, then it makes sense that mothers would report that they “receive better care” in their natal home, but it really is the belief system which is driving these results. Interviewees may not even considered this a driver because the belief system is so fundamental to them, and therefore the “better care” reason for them migrating is actually driven by these beliefs. I think this point is not sufficiently addressed. Even the authors believe that these beliefs are not driving the phenomena, I’d still like to be presented a reason why and I think it would be useful to have at least a brief discussion on this considering the importance. Was this mentioned at all in the interviews? If so, I would like it to be included.

Line 170: “women only represent 1.1% of women’s migration”

This sentence doesn’t make sense to me.

Line 203: “Two cadres of all-female CHWs work at the community-level delivering last mile services to pregnant, lactating women and infants – the Accredited Social Health Activist (ASHA) under National Health Mission and the Anganwadi Worker (AWW) under India’s flagship nutrition program, the Integrated Child Development Services [44,45].”

This sentence could be more clearly written.

Line 217: “we estimate at the impact of being in the natal home,”

I don’t think the ‘at’ is needed here.

Section 2.1: It would be useful to know how Madhya Pradesh and Bihar are different to one another, ideally using general descriptive statistics of the state, and how representative these states are of India as a whole. It would be useful to know a priori how we expect the results from these two states to differ.

Section 2.1: The authors should include reasons why these states were chosen for this analysis, because it is not clear as to why these specific states were chosen above all others.

Line 234: “then up to two AWCs/AWWs were sampled per village Then we randomly sampled up to eight mothers”

I think a full stop is needed here or change of character case.

Section 2.1.1: Is there no other source of selective sampling apart from apart from higher probability of sampling non-migrating mothers for your approach? Do some mothers "slip through the net" of the AWC/AWW system? If some mothers do slip through the net, then who are these likely to be and how would that impact your results and findings? The entire sample comes from those that engage with the AWC/AWW system. I think this is a major source of sample selection, and is a point not sufficiently addressed or consider.

Line 238,239: "interpreted this as indicating that by 6 months postpartum the vast majority of women have returned to their marital home."

Interpreted what exactly? Authors should be clearer. It's not clear on what you're interpreting or how you reach this conclusion.

Line 271: "We do this because the vast majority of women had met the Indian criteria of 4 or more, we wanted to highlight those that exceeded that threshold."

Are the authors able to show descriptive statistics of ANC visits, specifically what is meant by "vast majority of women"? It would be useful to see a granular distribution of this i.e. what % received 1 visit, what % received 2 visits, % that received 3 visits etc. because this seems like a peculiar discretisation decision to make, given that the guidelines are different to the authors categorisation. Your current analysis is "more than sufficient" vs. "sufficient or less", which is a strange discretisation in my opinion. "insufficient" vs. "sufficient" ANC visits would be more of an obvious discretisation, and your deviation from this is not well supported. It is a little strange that the reason for the discretisation is not supported with empirical evidence in the paper i.e. distribution of visits, so that the reader can see for themselves what "vast majority" means, and this would make the discretisation more convincing. Simply presenting whether received "Received 5 or more ANC visits" is not a sufficient descriptive statistic to justify this discretisation decision.

Section 2.1.3: Are all the independent variables estimates jointly in the same regression? I presume so, but it does not say explicitly.

Line 365: "Working outside of the home is associated with increased odds of returning to the natal home in Madhya Pradesh but reduces the odds of returning home in Bihar."

This is an example of a results which is perplexing, and if descriptive statistics and further background was presented for each state, then reader would be more prepared to understand these results. This happens a few times in the paper, and it's difficult to understand given that there is limited background on each state.

Line 410: "About 30% of women who returned home and those did not return home received 5 or more ANC visits (Table 5)"

This sentence could be written more clearly, because it doesn't make much sense to me.

Line 414: “Similar analyses for fewer ANC visits showed no effect (not shown).”

I think this is simply the inverse of “receiving 5 or more ANC visits”. How would these results be different to the ones from “receiving 5 or more ANC visits”, apart from the change in sign. It is not clear what this adds to your results.

Line 424,425: “multinomial models show no difference between private and public or home (also not shown).”

Even if they are not statistically significant, I would prefer if these results were not supressed and instead reported in the paper alongside the other results.

Line 435: Are the qualitative results in contradiction to the empirical ones? AWWs say that they can’t track mothers, and they find it harder to provide their services but being at natal address increasing odds of receiving an ANC visit. I realise these services are distinct, but if migrating mothers fall through the healthcare system, then how can it be that ANC visits are more likely to happen. It would be useful to have some discussion around this point, or to provide a little more background on the difference between AWWs and ANC services, because that might make these results more intuitive. These findings make me a little confused, and it may be insufficient background. This point is partially addressed in the discussion, but the authors don’t provide any reason why this might be the case, and I think this is important because I conclude that it is some form of statistical bias in your estimates without a reasonable explanation.

Section 5, line 465: I think the number of this heading is incorrect.

Section 5: It would be useful to discuss the overall health consequences of childbirth migration. Presumably the migration itself could impact health of mother and baby negatively, due to the stresses involved with migration itself. On the other hand, better care is provided at the natal home. Authors should discuss the overall health consequences of temporary childbirth migration.

Section 5: The authors should discuss policy implications. Specifically, it would be useful to know what these results mean for prenatal and postpartum healthcare provision, and how these issues could be tackled.

Section 5.1: It would be very useful to have some background on the differences between these states when describing the data earlier, so that the readers know what to expect when it comes to these differences. I have made this point already but, it is not clear how these states differ substantively prior to this discussion, and how these states differ to the average Indian state.
