# Supplementary material for: Temporary childbirth migration and maternal health care in India
Source: PLoS One. 2024 Feb 8;19(2):e0292802. doi: 10.1371/journal.pone.0292802 (PMC10852266; doi:10.1371/journal.pone.0292802)
Supplement: Attachment — Submitted filename: Review_PlosOne_Round1_revised.pdf [file pone.0292802.s004.pdf]

Temporary Childbirth Migration and Maternal Healthcare in India

Overview: The paper addresses a very important and relevant research question related to migration of women due to childbirth in two states of India – Madhya Pradesh & Bihar). This phenomenon has been largely understudied and undocumented. The paper provides analysis (both quantitative & qualitative) related to three major things:

a) Prevalence of this phenomenon
b) Socio-economic determinants for this phenomenon
c) Quantity of healthcare received by mothers due to this phenomenon

While the paper describes the prevalence of this phenomenon quite clearly, I have concerns about the way sample was selected for subsequent analysis (see point 1 under major comments). The analysis will benefit from robustness checks described below which can make the results more believable. Lastly, the results are not consistent across states (for both determinants of migration (in Table 2) and healthcare received (in Table 6)), this apparent difference in results has been discussed qualitatively (in section 5.1) but have not been quantitatively supported.

Detailed Comments:

Major:

1) The results related to socio-economic determinants of temporary child birth migration (Tables 2) uses a sample which chooses women who are 6 months post-delivery (child age > 6m) however this sample selection leads to a substantial drop in observations. A robustness check designed around this cut-off (time elapsed period post-delivery) for sample selection will help in establishing that the results are not sensitive to a **particular cut-off that was chosen for analysis**. Based on discussion in section (lines 384 to 386; 355) it makes sense to choose a different cut-off which is 3 months or 1 month post-delivery.

2) The empirical analysis introduces many control variables however an important determinant in form of **district fixed effects** is missing. Since majority of women might be migrating within the same district (lines 343 to 346) hence it makes sense to use district fixed effects which accounts for time-invariant heterogeneity (health infrastructure in a district etc). Most of the major health programs focused on child and maternal health are implemented in a top-down fashion where districts submit their expected budgets to the health ministry, the funds are also allocated to a district. To a great extent implementation of health policies in India are run/directed by the district administration. So, a strong suggestion is to include these fixed effects in all analyses that have been carried out in this paper.

3) How many women (in each state) out of the total sampled women participated in temporary childbirth migration? The analysis can be complemented with a **selection model** wherein in the first stage decision to migrate is modelled and in the second stage healthcare outcomes are modelled.

4) The reasons provided for difference in results for the two states (results are present for MP but not for Bihar) remain unclear. These reasons should be elaborated further and if possible, mechanisms should be explored which explain the difference in results for the two states. Perhaps NFHS dataset (which is representative at district level) can be used to document this systematic difference in healthcare delivery in these two states (specifically in the 12 districts which are the focus of this study).

5) Table 6: Explores the relationship “Five or more ANC visits” and “Being at natal home during last month of pregnancy”. Shouldn’t the dependent variable be number of ANC visits during last month of pregnancy. Most of the ANC visits in this scenario (where a mother only migrated during last month of the pregnancy) would have already taken place in her marital home rather than natal home.

Minor:

Overall, the writing is lucid but, in some places, it will benefit from further elaboration/clarification.

- Line 526: "Slightly more women returned to their natal home in Bihar compared to Madhya Pradesh, *however the difference was of practically meaningful*" Does this mean that the difference between the states was statistically insignificant? If yes, then it contradicts the statement in line 538: "First, we see that more women return to their natal home in Bihar".
- Lines 607 to 610 provide a back of the envelope calculation for number of women who face disrupted perinatal care due to temporary childbirth migration. However, this is confusing and contradicts the main findings of the paper (for Madhya Pradesh) which show that *despite* temporary childbirth migration, women received *more* care. Even for Bihar the null result signifies no drop in the level of care. Hence, in light of the results of the paper (and conclusion as mentioned in abstract) can the authors claim that there has been a disruption in health care for mothers who participated in temporary childbirth migration?
- Add another very important contribution to the literature: many papers which use Demographic Health Survey (DHS) datasets focus on child health outcomes and relate them to exposure to pollution or shocks during month of birth but use residential location as the place where these shocks were observed (which may not be correct). In light of this study, an important factor is revealed to the researchers in form of substantial proportion of mothers migrating to their native place during month of birth of a child. Few studies which have used month of birth along with residential location include – Spears, Menon etc.

  *Brainerd, E., & Menon, N. (2014). Seasonal effects of water quality: The hidden costs of the Green Revolution to infant and child health in India. *Journal of Development Economics*, *107*, 49-64.

  * Spears, D., Dey, S., Chowdhury, S., Scovronick, N., Vyas, S., & Apte, J. (2019). The association of early-life exposure to ambient PM 2.5 and later-childhood height-for-age in India: an observational study. *Environmental Health*, *18*(1), 1-10.
- Line 351 – The figure about one-third women (32.4%) participating in temporary childbirth migration cannot be located in Figure 2 (as referenced in the manuscript).
- I am not very clear about propensity score matching mentioned in line 233 in section 2.1.1. Why was it used? What was the dependent variable?
- (This is optional) Can the authors include actual empirical model in the manuscript i.e. write the model equations which are used for analysis?
- Discrepancy in line 242? Number of observations mentioned as n = 3055 but this figure doesn't match with 782+1273.
- Tables 3, 4: Are the significance levels indicative of difference between states? Mention this detail in table footnotes.
- Table 5: Different significance definition mentioned in table footnotes. Please use uniform significance level definitions throughout the paper.
- Line 422: Should be "….more likely…" rather than "more like".
