# Supplementary material for: Temporary childbirth migration and maternal health care in India
Source: PLoS One. 2024 Feb 8;19(2):e0292802. doi: 10.1371/journal.pone.0292802 (PMC10852266; doi:10.1371/journal.pone.0292802)
Supplement: Attachment — Submitted filename: PlosOne_R2.pdf [file pone.0292802.s005.pdf]

Temporary Childbirth Migration and Maternal Healthcare in India

The authors have revised their manuscript based on suggestions, but I don't see the new results anywhere (although authors have added text related to the comments). At this stage I would like to request authors to **provide all the results requested in a separate file or the main draft** so that they can be assessed and properly included in the main draft.

Specifically, the following results should be provided and **complemented with a detailed discussion of the results** (i.e. how the coefficient of interest changes etc) and **models** which were used:

1) Replication of results in Table 2 with 3-month post-partum as the cut-off, 1-month post-partum as the cut-off and full-sample (where no cut-offs are used). Please provide explanation as to how the coefficient is stable despite changing the cut-offs which establishes the robustness of the result.

2) Show results where District fixed Effects have been used. Please add text which discusses how the estimates remain unchanged after the introduction of district fixed effects.

3) Provide results with **selection model**. Show results for both the first and second stage and provide a commentary on these results as well. Please provide exact empirical models/equations which were used to reduce any confusion, this will help in better understanding and will provide more clarity.
   Suggestion: The selection model is a superior model of analysis in this set-up so rather than treating it as a robustness/sensitivity check, the main results can be replaced by the results from the selection model.

4) For results provided in Table 6: the main explanatory variable should be changed to "number of months spent in the natal home during pregnancy" in light of the explanation provided by the authors. This new variable will correctly capture the relationship between length of stay at natal home and care received in form of ANC visits. The older variable "being at natal home during last month of pregnancy" will not correctly capture the total number of ANC visits tied to the natal home.

   For reference my previous comment and current authors response:
   "5) Table 6: Explores the relationship "Five or more ANC visits" and "Being at natal home during last month of pregnancy". Shouldn't the dependent variable be number of ANC visits during last month of pregnancy. Most of the ANC visits in this scenario (where a mother only migrated during last month of the pregnancy) would have already taken place in her marital home rather than natal home.

   >> Yes, we agree that some of these visits may have occurred before the woman migrated, however, what we know about care seeking in pregnancy in India (and elsewhere) is that few women have visits early in pregnancy, and the number of visits increases in later pregnancy. A different (and interesting) analysis could be to see if women who migrated early in pregnancy were more likely to have a visit in the first trimester (which is an important gap), however, we did not do so in this analysis because of sample size issues with the number of women migrating in early pregnancy and because we were interested in using a standard measure of appropriate/adequate ANC visits. We had the exposure be "being at the natal home in the last month of pregnancy" to make sure that we were comparing appropriate groups of women to each other. For example, if we looked at women who were at the natal home in month 6, they might not have had enough time to get the adequate number of visits. By looking at women in the last month of pregnancy we are able to ensure that all women had comparable time to have enough visits."
