# Supplementary material for: Temporary childbirth migration and maternal health care in India
Source: PLoS One. 2024 Feb 8;19(2):e0292802. doi: 10.1371/journal.pone.0292802 (PMC10852266; doi:10.1371/journal.pone.0292802)
Supplement: Attachment — Submitted filename: renamed_0b548.pdf [file pone.0292802.s007.pdf]

Temporary childbirth migration and maternal health care in India – Comments for Authors

Overall, I think this paper is interesting and does paper does contribute to the literature by documenting an under investigated phenomena. The paper does contribute a documentation of an under investigated phenomena which potentially has some profound implications for policy. The analysis, although limited in scope does appear to be a reasonable approach. The discussion and conclusion do follow from the results. However, the paper does not extend much beyond documenting this phenomenon. Given that the main contribution of the paper is to document the phenomena, it may be worthwhile emphasising why this is important to document and what implications these results have, especially for health outcomes.

I can see that documenting this phenomenon is potentially important for policymakers and for future research. For researchers, not knowing that mothers migrate during pregnancy or soon thereafter, is likely causing survey sampling issues because they migrate. Also, the authors make clear that many policies designed to support mothers during pregnancy are not designed to accommodate maternal migration, and therefore is important to document.

Overall, the authors have addressed reviewers' comments. I am happy with the changes that were made, and the authors have improved their responses, compared to last time. However, there are still a handful of comments that need addressing prior to publication. These are small details but are necessary for publication.

The authors state that three items were included in the appendix, however I see only 2 tables. Specifically, the item which was requested "Appendix 1: Sociodemographic Characteristics of women who migrated at all compared to women who did not migrate at any point" is not included and missing, although the caption is included. Authors need to include this table in the appendix.

In Appendix Table 3, it is unclear what is meant by "district-level variable" and I don't understand what the coefficient on this variable means. It was district fixed effects which were requested; therefore, I would not expect "district" to be included in the table with a coefficient. The reviewers requested a district fixed effect regression, and therefore the reviewers need to estimate this model and include this in the appendix. Usually, a district-

level fixed effect model would not include “district” as an additional variable with a coefficient in the table .

Table 6 is unreadable, as it spans outside of the range of the page, and therefore I couldn’t read it. Authors need to make sure that pages with large tables, such as these, are either landscape, or scaled so that they are readable.

This comment was not addressed:
“Reviewer 2’s comment: “Tables 3, 4: Are the significance levels indicative of difference between states? Mention this detail in table footnotes.”
In would usually prefer that tables be readable on their own, without the need for inspecting the text. In other words, full details of how the numbers in the table were calculated should be included in the footnotes. Putting “*** p<0.01, ** p<0.05, * p<0.1” does not tell the reader what test was done to reach that result, was it the difference in means between states, or was it the test of coefficients being different from zero.”

Changes were made to other tables, but not to Tables 3 and 4. Again, what do these stars denote? Is this the significance in a t-tests of differences in means? Whatever the test used, include in footnote exactly what test was done to get these p-values. Be precise about where these p-values come from. The authors need to include precise detail of what test of differences was done, and state that they are testing the difference between states, in the footnote of Tables 3 and 4.
