# Supplementary material for: Temporary childbirth migration and maternal health care in India
Source: PLoS One. 2024 Feb 8;19(2):e0292802. doi: 10.1371/journal.pone.0292802 (PMC10852266; doi:10.1371/journal.pone.0292802)
Supplement: Attachment — Submitted filename: Review_PlosOne_Round3_revised.pdf [file pone.0292802.s008.pdf]

<u>Temporary Childbirth Migration and Maternal Healthcare in India</u>

Authors have tried to address many of the issues which were raised in the previous rounds however many of these were not sufficiently addressed, the draft lacks clarity in multiple areas and requires significant amount of rework.

Comments which have been raised before:

Please note I follow the same numbering as the one in my previous review (Items 1 to 5)

1) **Different cut-off points** for analysis (Table 2): Authors have addressed this partially, but they have not shown results for 1-month/3-month post-partum cut-off. They mentioned possible bias in the results (I am unclear about their reasoning/argument) but it is still desirable to see these results even if they are not a part of the final draft but only provided to the reviewer as part of author's response. Looking at these results will convince us that the authors are not cherry picking which analysis to show.

2) **District Fixed Effects**: This comment has been addressed in Table 6 and has been incorporated *incorrectly* in Table 2 (results show one single variable "Districts" rather than dummy variables for each district).

3) **Selection problem:** [Table 2 & 6 results will be directly affected by this analysis]: This concern has been raised by both the reviewers. Even though I had explicitly requested the mathematical model (first stage – which models the binary decision to migrate and second stage which models the outcomes we are interested in) and description of models/coefficients, authors haven't provided these in detail and in fact some of description of the models is insufficient. Below is authors description of first stage (lines 358-359, 367-371):
"….generalized structural equation models to accommodate for potential selection effects into each temporary childbirth migration variable by sociodemographic characteristics."
"First stage models in joint analyses for temporary childbirth migration outcomes employed Poisson regression for the number of months spent in the natal home during the last trimester of pregnancy and logistic regression for natal home at time of childbirth for delivery analyses and natal home during the first month postpartum, also following above empirical models."

   - What was/were the variables used in the first stage which was/were not included in the second stage (refer to Heckman model)?
   - How come the first stage is poisson model in case of a selection model (as mentioned in lines 367-371)? Shouldn't it be a logistic model in all cases? [Please note that my original comment#4 in review round#2 was suggested when you were NOT using a selection model.]
   - Where are the results for first stage regression (in one of the many panels of Table 6?)? Where have they been discussed in detail in the manuscript?
   - The language is also quite confusing. I would suggest rewording this content for clarity.

4) **Outcome choice in Table 6**: First of all, this table in its current form in unreadable. The table doesn't fit the page, different panels have been thrown together without appropriate labelling, the text description is unclear. What is the sample size for this analysis?
   Outcomes chosen by authors for Table 6:
   Number of ANC visits – This was observed at which time-window, the last month of pregnancy (as you mentioned in your response)? Please mention this in brackets as well in the results section so that clarity is maintained.
   Place of delivery – This choice is fine as it doesn't depend on any particular choice of time window.
   Number of post-partum visits – Was this asked in the first month postpartum (as mentioned in line 311?)? Isn't that shorter/different from the Indian guideline which is meant for 42 days rather than a month (line 330)?

5) Similar to item #4.

Another item which is still unaddressed (was raised by Reviewer#1 in the last round and previously by me as well) is describing WHY results are different for the two states. Authors describe the state of ICDS in these two states and mention few other statistics but as Reviewer#2 mentioned this apparent difference has still not been rationalized for the reader.

The issues related to empirical analysis/writing/layout/lack of clarity remain in the manuscript. Particularly, the lack of clarity/convincing arguments is a recurring issue in the manuscript where the content doesn't flow/connect across sections and thus creates confusion.

<u>Minor Comments:</u>

1) Reference # 5 & 52 are same, retain only one.
2) Line 250 mentions "Village matched" this creates confusion as the content of the companion paper is not a part of this draft. Either describe this approach in a footnote or choose alternate wording.
3) Line 239-240: "We hypothesize that natal home migration will lead to a disruption in the continuum of care and therefore lower access and utilization of services (Figure 1)." Literature provided by the authors suggests a movement in either direction, how was this particular hypothesis (i.e. disruption) selected?
4) Line 284: Replace "12m" with less than 12 months age
5) Reference to Appendix items 1 & 3 missing in draft.
6) Table notes should be expanded, at this stage it is not possible to read the tables independent of the text. Describe the model used, dependent, key independent variable/s, mention what * represent i.e. what is the hypothesis being tested.
7) Mention in text and table what is IQR and IRR (along with their full form).
8) The legend description of mathematical equation is incorrect. It should be β_i and not $\beta_{1-i.}$
