# Supplementary material for: Temporary childbirth migration and maternal health care in India
Source: PLoS One. 2024 Feb 8;19(2):e0292802. doi: 10.1371/journal.pone.0292802 (PMC10852266; doi:10.1371/journal.pone.0292802)
Supplement: Attachment — Submitted filename: Response to Reviewers.docx [file pone.0292802.s006.docx]

March 19, 2022

To the editors and reviewers,

Thank you again for the opportunity to revise and resubmit this paper, and for your very thoughtful and detailed comments. We have worked to again revise this paper, being more careful to include all new models and address the reviewer comments.

Please find a point by point response below, and let us know if you have additional questions,

Thank you again for your time and support,

Nadia Diamond-Smith

Reviewer #1: This paper documents a seemingly under-documented phenomena, that being maternal migration during pregnancy, and post-pregnancy, or as the authors name it “Temporary Childbirth Migration”. The authors use mixed methods to describe the phenomena of migrating during pregnancy or postpartum in two states in India.
Comments that myself and previous reviewer made in our last review were somewhat addressed, but there are still several comments which were not addressed sufficiently. My major concern is the continued (and increased) reference to analyses which was done and is never shown to the reader. I understand the authors preference for a compact paper, but these results could be presented in an appendix or supplementary material. I do not like the reference to analysis the reader is unable to access.
Below I have outlined comments which I consider to be major and more minor comments. I think these comments require addressing prior to publication.


MAJOR COMMENTS
-Section 2.1: The authors should include reasons why these states were chosen for this analysis, because it is not clear as to why these specific states were chosen above all others.
This point was not sufficiently addressed. State how the availability of comparable control districts was evaluated, was this done statistically? Also state what the reasoning was for the Ministry of Woman and Child Development and the funding agency to suggest these districts. The question of why these districts were chosen does pose some questions about credibility, and I think the authors need to be more explicit in their decision for choosing these states. The including of the comments they make are welcome, but are not sufficient.

We have added more details to clarify and to increase the credibility of our research but adding the complete study design already covered in the protocol would make the paper lengthy. As such, we also have added the protocol paper as a supplementary material and request the readers to read that in detail.

- Section 2.1.1: Is there no other source of selective sampling apart from apart from higher probability of sampling non-migrating mothers for your approach? Do some mothers “slip through the net” of the AWC/AWW system? If some mothers do slip through the net, then who are these likely to be and how would that impact your results and findings? The entire sample comes from those that engage with the AWC/AWW system. I think this is a major source of sample selection, and is a point not sufficiently addressed or consider.
This point was not addressed at all. If, as the paper claims, the hypothesis is that those temporarily migrating are less likely to engage in the AWC/AWW system, then there is a major selection problem here, and this induces a major source of bias in your estimates. Given that a key contribution of the paper is to sample mothers who are usually missed in surveys, it is worth stating specifically how this paper addresses those limitations of previous work. How are these individuals recruited given that they usually fall through the net? What do you do to mitigate this?

Yes, this is a limitation of this study. We have extended our discussion of this limitation in the paper as such “Our data also come from women who are registered at Anganwadi Centres, so the results are only generalizable to women from these districts who are registered in the ICDS system. The NFHS-4 in India, which was collected around the time of this study, found that 65% of women who gave birth in the last 5 years in Bihar and 81% in Madhya Pradesh were registered (5). Thus these findings can only be interpreted as describing maternal migration and associated impacts on care among a population of women who are already in the health care system during their pregnancies. Women who are never registered in pregnancy may be unlikely to receive care at all, and thus might face no impacts of migrating during pregnancy (since they were never seeking care). On the other hand, women who do not register in their husband’s home may migrate and somehow seek care in their natal home. More research is needed on this population.”

- Lines 306-314: I made a similar comment in previous review, but it was not sufficiently addressed. This feels a little like you’re picking the goalposts that suit your results, either that or the language makes it confusing. The WHO recommend 8 ANC visits, of which how many should be in the last month? The Indian government recommend 4 visits, of which how many should be in the last month? Is the measure of 5+ ANC visits for the last month only, or entire pregnancy? The choice of dependent variable should at least align with one of these 4 guidelines. If your outcome variable is for the final month, then it should exactly align with WHO’s or the Indian governments recommendations for the final month, not for the entire pregnancy. If there is no guideline for the final month, but you want your dependent variable to be ANC visits in final month, then it should be a count variable, not a binary one. This analysis is confusing, and I’m not sure whether your outcome aligns with one of the guidelines. I don’t think a reasonable argument to make is that the Indian guideline for entire pregnancy is 4 visits, so we look to see if mothers had over 4 visits in the final month, because this is not what the guideline says from my understanding. It may be that I do not fully understand the guidelines, in which case the writing needs to be tidied up a little.

Thank you for this comment. We agree that the guidelines are confusing and it is hard to know what the marker of appropriate ANC is given that the WHO and the Indian guidelines differ. We discussed these comments as a team, and decided to present the analysis as a continuous variable (number of ANC visits) so that we are not adding a value judgement to the appropriate number of visits. We have thus changed the table and the discussion in the text. We kept the figure in that shows number of ANC visits to give the reader more understanding of the situation with ANC visits in this population.

- I agree with Reviewer 2’s major comment 2 from previous review. Clustering does not address the issue that reviewer 2 raises and I find it a little difficult to believe that the results would be the same if fixed effects were included. I understand the authors do not want to include too many tables, but they may consider an appendix, because there are several analyses which are referred to throughout the text, but the reader has no access to them.
This comment is also applicable to Reviewer 2’s major comment 1 and 3. The constant reference to analysis that was done and a conclusion of “the results were the same” does, unfortunately, question the credibility of the analysis.
Indeed, this is a more general point that there are several instances throughout the paper that refer to additional analysis which the reader is not able to access. I would suggest this needs remedying and including an appendix would be a reasonable approach, given the authors preference for a more compact paper.

We apologize for not adding these additional analyses earlier. We have added 3 new appendices which show the additional sensitivity analysis that were conducted. We have added district fixed effects in our models.

- Reviewer 2’s previous comment: “I am not very clear about propensity score matching mentioned in line 233 in section 2.1.1. Why was it used? What was the dependent variable?”
Although this was improved, I think the authors need to be marginally more explicit. Presumably the nearest neighbour matching was done, and some measure of error was used to choose the districts. But it’s still not clear why 852 villages were chosen, what was the criteria? Was it that 852 villages met your pre-set criteria, or was it that you wanted 852 villages and these ones were the best fitting ones?

We have elaborated more on the sample size calculations and how the number of villages were calculated. More details on the propensity score matching approach are available in our protocol paper made available as supplementary material for the reader.

MINOR COMMENTS
- I still find it a little peculiar that although there is at least anecdotal evidence of the ritual pollution, none of the survey respondents state that this was a reason for leaving. Although beyond the scope of this paper I think this is an important avenue for future research and exploration.

We agree that more research should be done on this. It is possible that some of the rationale behind the responses given in the quantitative survey could be interpreted as being because of ritual pollution (for example, “It is the cultural and social norm” or “Husband’s family asked me to go”).

- Previous review comment: “Line 365: “Working outside of the home is associated with increased odds of returning to the natal home in Madhya Pradesh but reduces the odds of returning home in Bihar.”
My preference is for these differences to be discussed and rationalised, because at present it’s difficult to draw conclusions (esp. for policy makers) because the effect is exactly in the opposite direction for each state. I would prefer that these differences be explained to the reader, because it is perplexing; without some explanation the reader is unsure of what conclusions to draw. The state specific description doesn’t really help explain this result. I would prefer the authors make it easier for the reader to understand the strange result.

There are many differences between the states which are somewhat complexing, we agree. We have removed a few of the sentences about state differences since that is confusing in places. While the results are the appropriate place for us to be interpreting these differences, in the discussion we do have a section talking about broad differences between the states, which we hope provides the reader with some information about why these differences exist. Future research could explore contextual differences in more depth.

- It may be worth including a table similar to “Table 1: Demographics of sample” but instead by whether temporary migrated or not.

Thank you, we had felt that Table 2 explored the differences in the socio-demographics by different migration statuses, but we have included the table that you requested as an appendix and will let the editors decide if they would like both of these in the paper.

- Reviewer 2’s comment: “Tables 3, 4: Are the significance levels indicative of difference between states? Mention this detail in table footnotes.”
In would usually prefer that tables be readable on their own, without the need for inspecting the text. In other words, full details of how the numbers in the table were calculated should be included in the footnotes. Putting “*** p<0.01, ** p<0.05, * p<0.1” does not tell the reader what test was done to reach that result, was it the difference in means between states, or was it the test of coefficients being different from zero.

Thank you, we have clarified that this table shows the results of logistic regression models by adding this to the title, along with “*(Odds Ratios, 95% confidence intervals)”*

Reviewer #2: Temporary Childbirth Migration and Maternal Healthcare in India

The authors have revised their manuscript based on suggestions, but I don’t see the new results anywhere (although authors have added text related to the comments). At this stage I would like to request authors to provide all the results requested in a separate file or the main draft so that they can be assessed and properly included in the main draft.
Specifically, the following results should be provided and complemented with a detailed discussion of the results (i.e. how the coefficient of interest changes etc) and models which were used:

1) Replication of results in Table 2 with 3-month post-partum as the cut-off, 1-month post-partum as the cut-off and full-sample (where no cut-offs are used). Please provide explanation as to how the coefficient is stable despite changing the cut-offs which establishes the robustness of the result.

We have added an appendix with the results of Table 2 for any migration run for the full model. We have clarified in our text that while there are small differences, they do not change the direction or interpretation of data (“no meaningful differences in the interpretation of the findings (Appendix 2).” We do not think that it makes sense to show the analyses for the other cut offs (3 months, 1 month) because many women are still at their natal home during this time, and thus not captured in our data, and thus just showing these results would be biased. Showing all the data combined shows that the smaller sample we selected to focus on for the rest of the analyses which we feel captures most of the return migrants is a reasonable choice, even though it restricts our sample size somewhat.

2) Show results where District fixed Effects have been used. Please add text which discusses how the estimates remain unchanged after the introduction of district fixed effects.

We have added district fixed effects to table 2 data for any migration. As can be seen the addition of this to the model does not change the magnitude of the associations meaningfully or direction of any associations. We think that because we cluster at the village level that this might already account for given correlation between clusters and villages and districts.

3) Provide results with selection model. Show results for both the first and second stage and provide a commentary on these results as well. Please provide exact empirical models/equations which were used to reduce any confusion, this will help in better understanding and will provide more clarity.
Suggestion: The selection model is a superior model of analysis in this set-up so rather than treating it as a robustness/sensitivity check, the main results can be replaced by the results from the selection model.

We have revised our primary analyses for the impact of temporary childbirth migration on our four health-related outcomes to selection models, including results for both the first and second stage.

4) For results provided in Table 6: the main explanatory variable should be changed to “number of months spent in the natal home during pregnancy” in light of the explanation provided by the authors. This new variable will correctly capture the relationship between length of stay at natal home and care received in form of ANC visits. The older variable “being at natal home during last month of pregnancy” will not correctly capture the total number of ANC visits tied to the natal home.

Apologies if our last response was not clear and led to more confusion. Unfortunately we did not collect data on each month of pregnancy and where the woman was, instead, as can be seen in Figure 3, months 0-3 and 4-6 were clumped together, and thus we only know if they spent any of those months at their natal home and we could not create a total number of months variable. The WHO recommendations are that 3 of the 8 ANC visits are supposed to be in the last month of pregnancy, and 2 in the second to last month. Based on the reviewer’s feedback, we created a new variable that summed the number of months that a woman was out of the last trimester (months 7, 8 or 9) and ran new models, which we have now included in the paper. These results do not change our interpretation of any of the relationships, but may be a better measure of the exposure. Thank you for this comment.

For reference my previous comment and current authors response:
“5) Table 6: Explores the relationship “Five or more ANC visits” and “Being at natal home during last month of pregnancy”. Shouldn’t the dependent variable be number of ANC visits during last month of pregnancy. Most of the ANC visits in this scenario (where a mother only migrated during last month of the pregnancy) would have already taken place in her marital home rather than natal home.

Yes, we agree that some of these visits may have occurred before the woman migrated, however, what we know about care seeking in pregnancy in India (and elsewhere) is that few women have visits early in pregnancy, and the number of visits increases in later pregnancy. A different (and interesting) analysis could be to see if women who migrated early in pregnancy were more likely to have a visit in the first trimester (which is an important gap), however, we did not do so in this analysis because of sample size issues with the number of women migrating in early pregnancy and because we were interested in using a standard measure of appropriate/adequate ANC visits. We had the exposure be “being at the natal home in the last month of pregnancy” to make sure that we were comparing appropriate groups of women to each other. For example, if we looked at women who were at the natal home in month 6, they might not have had enough time to get the adequate number of visits. By looking at women in the last month of pregnancy we are able to ensure that all women had comparable time to have enough visits.”
