# Supplementary material for: Temporary childbirth migration and maternal health care in India
Source: PLoS One. 2024 Feb 8;19(2):e0292802. doi: 10.1371/journal.pone.0292802 (PMC10852266; doi:10.1371/journal.pone.0292802)
Supplement: Attachment — Submitted filename: Response to Reviewers_R4_20230530.docx [file pone.0292802.s010.docx]

May 31, 2023

To the editor and reviewers,

Thank you for the opportunity to revise and resubmit this paper, and apologies for the very delayed response time. We have very carefully detailed our response to the reviewers below. We would like to note that we involved a PhD-level biostatistician to assist us with reviewing and revising analysis and its description, due to the back and forth that we have had over the last few rounds of reviews. We carefully considered the feedback and have responded below to the very best of our ability.

Reviewer comments in bold, our responses in regular text. Please let us know if you have additional feedback.

Thank you again for your time and feedback,

Nadia Diamond-Smith, on behalf of my co-authors

**Reviewer #1: Major Comments

The selection model comments made by Reviewer 2 (comment 3 in round 3) has not been addressed. My interpretation of the back-and-forth between the review and author is that the authors do not understand the comments being made, either that, or the discussion in the paper text are not sufficiently clear to describe the analysis. The problem that Reviewer 2 is outlining is that the second stage includes only those individuals that migrated, which is a non-random sample of the population, and including only migrants induces bias in your analysis from this non-random selection. The purpose of the selection model is to reduce the bias caused by the analysis of a non-randomly selected sample. The procedure is as follows (for heckman selection):
1) Estimate the probability of being in the sample (using all observations including those that don’t appear in your secondary analysis) using a probit,
2) Include the inverse mills ratio -- which is calculated using the probabilities from the first stage -- in your second stage equation. I believe your second stage equation are your main results, however I’m not sure if they are sample selection corrected by including the inverse mills ratio or equivalent.

There are alternatives to the heckman selection, however sample selection models are usually two stage and the details of each stage should be made explicitly clear in the text. It is also usually the case that authors present estimates from the first stage in their analysis. Review 2 has asked that a sample selection model be carried out and that the first stage results are presented for the reader to see.

In response to authors’ comment: You can combine both a Heckman selection model with a Poisson regression in the second stage with the ‘heckpoisson’ command in Stata.

It is possible that we have misunderstood your model – which is why I usually prefer mathematical equations to be used when discussing quantitative analysis, as it makes it easier for the reader to understand the authors approach – however this suggests that the discussion of the analysis requires improvement, so that the reader can better understand what is being done. If a sample selection model is not required, because the sample is randomly selected, then this should be better stated in the text.**

We appreciate both reviewers’ guidance that further details are needed to clarify our modeling approach and our rationale for this analysis. In presenting our response, we would first like to share the back and forth between the authors and reviewers across the four rounds of review that this paper has undergone for context. We then describe the edits which have been made since our prior version.

- In our **first round** of reviewer comments (September 2021), Reviewer 2, Comment 3 suggested a selection model as follows:

*Reviewer feedback: 3) How many women (in each state) out of the total sampled women participated in temporary childbirth migration? The analysis can be complemented with a selection model wherein in the first stage decision to migrate is modelled and in the second stage healthcare outcomes are modelled.*

*Author’s response: A total of 2,921 women overall participated in any temporary childbirth migration, 1,753 in Bihar and 1,168 in Madhya Pradesh. We have added these numbers to the text.*

*We appreciate the reviewer’s suggestion for extending our analysis and have developed joint models incorporating selection into temporary childbirth selection for each of our outcomes of interest using a generalized structural equation modeling approach. We ran these models and there were no changes in the relationships between temporary childbirth migration and the outcomes, and thus we have not changed our models in the paper. We have however added that we ran this as a sensitivity analysis in the paper itself “We also ran joint models incorporating selection into temporary childbirth selection for each of our outcomes of interest using a generalized structural equation modeling approach as another sensitivity analysis. These models did not differ substantially from the models presented and thus are not shown.”*

- In our **second round** of reviewer comments (March 2022), Reviewer 2 Comment 3 relates to the selection model, as follows:

*Reviewer feedback: 3) Provide results with selection model. Show results for both the first and second stage and provide a commentary on these results as well. Please provide exact empirical models/equations which were used to reduce any confusion, this will help in better understanding and will provide more clarity.
Suggestion: The selection model is a superior model of analysis in this set-up so rather than treating it as a robustness/sensitivity check, the main results can be replaced by the results from the selection model.

Author’s response: We have revised our primary analyses for the impact of temporary childbirth migration on our four health-related outcomes to selection models, including results for both the first and second stage.*

- In our **third round** of reviewer comments (September 2022), Reviewed 1 Comment 3 relates to the selection model, as follows:

*Reviewer feedback: 3) Selection problem: [Table 2 & 6 results will be directly affected by this analysis]: This concern has been raised by both the reviewers. Even though I had explicitly requested the mathematical model (first stage – which models the binary decision to migrate and second stage which models the outcomes we are interested in) and description of models/coefficients, authors haven’t provided these in detail and in fact some of description of the models is insufficient. Below is authors description of first stage (lines 358-359, 367- 371): “….generalized structural equation models to accommodate for potential selection effects into each temporary childbirth migration variable by sociodemographic characteristics.” “First stage models in joint analyses for temporary childbirth migration outcomes employed Poisson regression for the number of months spent in the natal home during the last trimester of pregnancy and logistic regression for natal home at time of childbirth for delivery analyses and natal home during the first month postpartum, also following above empirical models.” - What was/were the variables used in the first stage which was/were not included in the second stage (refer to Heckman model)? - How come the first stage is poisson model in case of a selection model (as mentioned in lines 367-371)? Shouldn’t it be a logistic model in all cases? [Please note that my original comment#4 in review round#2 was suggested when you were NOT using a selection model.] - Where are the results for first stage regression (in one of the many panels of Table 6?)? Where have they been discussed in detail in the manuscript? - The language is also quite confusing. I would suggest rewording this content for clarity.*

*Author’s response: We appreciated the reviewer’s recommendation to incorporate our two-stage analysis with a joint modeling approach and have operationalized this analysis within a generalized structural equation modeling (SEM) approach. This broader approach was selected as it allows for us to model our count and binary outcomes appropriately through the use of Poisson and logistic regression models, respectively. While the Heckman selection model is a special case of generalized SEM modeling, this modeling approach is limited in that it cannot accommodate non-binary outcomes and its application is limited to selection due to censoring/non-observation of data. We have also added further detail to the methods section and results section as requested by the reviewer.*

In response to the reviewers’ feedback, we have added additional explanatory text to the paper to clarify that our analysis was not designed to accommodate for selection in the sense of informative missing data, which is a traditional use of the Heckman model.^1^ Instead, we used it to accommodate for bias that may be due to unobserved factors that jointly influence both migration and the health outcomes. In acknowledgement of this complicated relationship and to reduce the likelihood of bias within our model of temporary childbirth migration and outcomes, we employ a joint modeling strategy to simultaneously answer the following research questions:

- First: What is the influence of sociodemographic characteristics (predictors) on temporary childbirth migration (outcome)?
- Second: What is the influence of temporary childbirth migration (primary predictor) and sociodemographic factors (secondary predictors) on outcomes: number of antenatal care visits, private (vs. other) facility delivery, and postpartum health check (vs. none), after controlling for the direct relationship between sociodemographic factors and temporary childbirth migration.

We implement this analytic approach within Stata’s generalized structural equation modeling (GSEM) procedure which accommodates our unique combination of endogenous covariates, hierarchical data structure, and outcome structures (i.e., both binary and count data).^2^ In re-examining our analysis based on the reviewer comments we identified an error in our statistical analysis code which excluded the individual level random effect linking the joint models that accommodate selection bias. This adjustment necessitated a revision to our modeling approach due to some challenges fitting these complex models with multiple levels of random effects and correlated error terms. To simplify our models, we moved district from a random effect to a fixed effect while maintaining village as a random effect, and we adjusted the approach used for covariate inclusion from an a priori strategy to based on minimum threshold of association of p<0.1 in unadjusted models. Finally, we monitored for evidence of selection within these models, and for models where selection was estimated to be 0, we exclude the individual selection parameter from the final model presented. This occurred for two models: 1) outcome of any facility delivery for Bihar and 2) private facility delivery for MP and is noted in the methods sections as well as under the table.

Compared to our prior analyses, the revisions presented within this resubmission were quite similar. Our interpretation of the findings on the impact of temporary childbirth migration (number of months at natal home in 3^rd^ trimester) on the number of antenatal care visits achieved for both Bihar and Madhya Pradesh did not change from our prior analysis (Table 7). Similarly, our analyses on the odds of postpartum health check by being at the natal home postpartum were qualitatively similar in our revised analyses (Table 7). There was one exception. Our analysis of the impact of temporary childbirth migration (at natal home during childbirth) on private facility delivery was similar to our prior analysis for Madhya Pradesh but different for Bihar (Table 7). Our prior analysis identified a 55% increased odds of private facility delivery with being at the natal home during childbirth which was borderline statistically significant, our revised analysis reflected a non-statistically significant decrease with a large confidence interval.

**Minor Comments

Table 1 goes off the page. Reformat for publication.**

Edited

**Stars representing results of statistical tests are a little odd. In table 6 there appears to have been two tests conducted, however I’d usually prefer different tests to be presented in different tables. My suggestion is to have two separate tables those being: Differences between states and the corresponding test (i.e. column for each state), and separately, between migration status and corresponding test (i.e. column for each migration status). The current presentation is a little peculiar.**

Thank you for this feedback, we have split this into 2 tables as you suggested and agree that this is much clearer. They are now labeled Table 6a and Table 6b.

**Looks like the fixed-effect analysis is consistent with the main results, I suggest mentioning that they are consistent in the main text.**

Please see our response to the reviewer’s earlier comment regarding selection bias where we note that we have revised our modeling strategy to include district fixed effects instead of random, given model complexity.

**Reviewer #2:**

**Authors have indeed addressed many of the comments from the last round. The presentation of tables/tables has also significantly improved, making it easier to read them without the main text. I will highlight areas/items which still need some work, some of the comments carry forward from the previous round (as they have been partially addressed) but I must highlight that all of the MINOR comments and MANY of the MAJOR comments have been handled in the current version.**

Thank you so much for this feedback. We continue to be extremely grateful to our reviewers for their dedication to improving our paper through ongoing thoughtful and detail-oriented review. We hope that in this fourth revision, we have satisfactorily resolved all major reviewers’ concerns.

**Comments:
1) ESSENTIAL/MAJOR
a) Selection problem: The mixed effects generalized structural model is used to address this in the current version of the manuscript. This approach uses a joint analysis for temporary child migration (a count or a binary variable) and health outcome (number of ANC visits, private delivery, postpartum care etc). The sociodemographic characteristics being used for modelling both the variables are identical. I still fail to understand how in absense of an exogenous variable this model is able to address the selection problem. The manuscript is also silent about this, and again this is something that I have raised before. The name of a model used doesn't automatically fill-in the gap about understanding HOW that model helps in addressing an issue (selection in this case).**

Our revised models accommodate selection through explicitly incorporating correlated errors terms across the two models. Please see other information regarding the revisions made to our models in our response to reviewer 1’s first comment.

**b) District Fixed Effects: This is again a comment which has been raised before but has been left unaddressed. Authors instead use a mixed effects model (incorporating for hierarchical structure of villages embedded in districts). However, clustering is not a perfect substitute for fixed effects. The idea is not to view (as authors responded in their letter) "district-specific differences in relationships" but rather to explicitly account for time invariant district specific characteristics and to see whether that changes the main relationships being studied in this paper. Having no transparency when it comes to these set of results does make me uncomfortable, since it should be pretty straight forward thing to implement.**

With recent modeling adaptations for our selection models incorporating district fixed effects (table 7), we have adapted table 3 to include district fixed effects for consistency. We have added some language to the results and discussion section noting some district-specific differences in migration behavior and outcomes and have added language to the discussion section referring to this as an area of interest for future research, as follows: “Indeed, our fixed-effects analyses identified some differences in migration behaviors and outcomes within district-specific comparisons which suggests that future research should further explore the role of district-specific contextual variables.” (p29)

**c) The current write up of Section 2.1.3 Analysis needs some more work. Equations for different outcomes should use different notations rather than identical ones which creates confusion. Also not all subscripts have been defined, what is "j" in u0J (line 362)? What is "j" again in equation 2? what is Xij (line 365)? Beta1 as mentioned in line 365 is not temporary childbirth migration (TCM) dose but rather the impact (coefficient) of TCM on health outcomes. A line describing why we need cluster random effects will also be helpful.**

Thanks for your feedback. We have expanded our explanations in this section to ensure that each parameter is clearly explained and we note the multi-level nature of our data to clarify the use of random effects to accommodate clustering.

**2) NEED MORE DISCUSSION / CHANGES**

**a) Why do results not hold for postpartum care? Table 7 presents these results and discussion section mentions this without any supporting discussion.**

Please see the paragraph starting on line 608 “Our findings that postnatal care (PNC) is not impacted by location similarly contradict the perspectives expressed by the AWWs, who expressed difficulties in identifying and providing care to women temporarily in their natal homes. It is possible that cultural practices of women staying in their homes for extended periods of time (mostly up to 40 days) in the postnatal period contributes to this, making it harder for AWWs to access them or even know of their existence, regardless of whether they migrated or not. Also, PNC received by mothers within 48 hours after delivery is already low (57% and 42% in Madhya Pradesh and Bihar respectively) and additional PNC is infrequent in general, perhaps because it is not highly prioritized by the government system or families, and therefore any difference might be hard to detect ^3^. Prospective designs and more nuanced quantitative and qualitative data are needed to disentangle these findings.”

**b) Table 6 - how do you read this table? There are stars in all columns for few rows.**

Thank you for this feedback, we have split this into 2 tables as you suggested and agree that this is much clearer. They are now labeled Table 6a and Table 6b.

**3) MINOR
a) Table 2 - how can the total on top of the column be calculated from rows below? Can you describe how the total for Madhya Pradesh (1296) be derived from rows below?**

The top column is the total number of women in each state, so adding the numbers below will not sum to that number. However, it is the denominator used to calculate the percentages. For example, in the first row, 782 women migrated at all, which is 42.3% of the full sample (782/1849=42.3%)

**b) Line 329-330 menion "As can be seen in Figure 2 most women in India do not meet the Indian guidelines, and only 25% receive more than 4 ANC visits." Figure 2 is about percentage of women indulging in TCM and not about ANC visits.**

Apologies, we had removed the wrong figure in a previous revision round, we have replaced this.

**c) Table 1 doesn't fit the page. Similar to other wide tables use widescape instead of portrait for this table**

Edited

**d) Couple of formatting errors (please carefully go through text again to be sure about any remaining errors like these):**

Thank you, we have carefully read through our text again and have made some additional edits.

**i) Line 101 insert space before (9,10).**

Added

**ii) Line 245 change to "...quantify the impact of Temporary Childbirth Migration on health service use"**

Edited

**iii) Line 253, change to "(52)."**

Edited

**iv) Line 255 insert space before bracket**

Edited

**v) Line 275-277: I can't understand the text currently. Perhaps remove or edit to improve readability.**

We have edited this section to read as below, we have also removed some of the superfluous information which was not needed for a reader to understand this sample for this specific analysis.

“1200 respondents from 200 villages in each arm from each state were needed to detect a difference of 5-9 percentage points in the child health outcomes of interest in the parent study (discussed above) from the counterfactual levels between 10-50 percent with an intra-cluster correlation coefficient between 0.15—0.30 ^4^”

**vi) All places where beta1-k is mentioned should have the k in subscript rather than normal text.**

We thank the reviewer for their identification of this typographical error and have corrected this across the methods section.

**vii) Line 573: correct to "...play out"**

Edited
