# Supplementary material for: Temporary childbirth migration and maternal health care in India
Source: PLoS One. 2024 Feb 8;19(2):e0292802. doi: 10.1371/journal.pone.0292802 (PMC10852266; doi:10.1371/journal.pone.0292802)
Supplement: Attachment — Submitted filename: Response to reviewer comments R3_1Sept22.docx [file pone.0292802.s009.docx]

September 1, 2022

To the reviewers and editors of PlosOne,

Thank you once again for your very thoughtful and detailed feedback on this manuscript. We greatly appreciate the energy you have put into helping us make this a strong and methodologically sound paper. Below we address each comment point-by-point.

Thank you for your time,

Nadia Diamond-Smith

Temporary Childbirth Migration and Maternal Healthcare in India

Authors have tried to address many of the issues which were raised in the previous rounds however many of these were not sufficiently addressed, the draft lacks clarity in multiple areas and requires significant amount of rework. Comments which have been raised before: Please note I follow the same numbering as the one in my previous review (Items 1 to 5)

1) Different cut-off points for analysis (Table 2): Authors have addressed this partially, but they have not shown results for 1-month/3-month post-partum cut-off. They mentioned possible bias in the results (I am unclear about their reasoning/argument) but it is still desirable to see these results even if they are not a part of the final draft but only provided to the reviewer as part of author’s response. Looking at these results will convince us that the authors are not cherry picking which analysis to show.

We apologize for any continued confusion and concern regarding our analysis. However, we think that the reviewer’s comment is due to confusion about our analytic sample rationale. We have revised our analysis so that we use a consistent analytic sample throughout the paper of women 6-12 months postpartum. We maintain our rationale for the selection of women 6 or more months postpartum in the methods section, describing that this is to ensure that our sample is representative of all postpartum women, including those who stayed longer at their natal home. If we were to define our sample using a 1 month or 3 month postpartum cutoff, our sample would be biased towards women who stay a shorter period, do not migrate, or some other factors. However, by focusing on women who are back at their marital home by 6 months postpartum, we have data on women who returned earlier, but we also have data on women who returned later, thereby reducing any bias towards women with short stays or who do not migrate at all.

2) District Fixed Effects: This comment has been addressed in Table 6 and has been incorporated incorrectly in Table 2 (results show one single variable “Districts” rather than dummy variables for each district).

We have revised our regression models to a mixed-effects modeling strategy incorporating the two hierarchical effects, village and district levels, to accommodate for clustering. A population-averaged model better fits our analytic interest in the role of district within the current analysis, which is to accommodate for our sampling structure for each state, and not to explicitly model district-specific differences in relationships. Our text and tables have been modified to the new analysis.

3) Selection problem: [Table 2 & 6 results will be directly affected by this analysis]: This concern has been raised by both the reviewers. Even though I had explicitly requested the mathematical model (first stage – which models the binary decision to migrate and second stage which models the outcomes we are interested in) and description of models/coefficients, authors haven’t provided these in detail and in fact some of description of the models is insufficient. Below is authors description of first stage (lines 358-359, 367- 371): “….generalized structural equation models to accommodate for potential selection effects into each temporary childbirth migration variable by sociodemographic characteristics.” “First stage models in joint analyses for temporary childbirth migration outcomes employed Poisson regression for the number of months spent in the natal home during the last trimester of pregnancy and logistic regression for natal home at time of childbirth for delivery analyses and natal home during the first month postpartum, also following above empirical models.” - What was/were the variables used in the first stage which was/were not included in the second stage (refer to Heckman model)? - How come the first stage is poisson model in case of a selection model (as mentioned in lines 367-371)? Shouldn’t it be a logistic model in all cases? [Please note that my original comment#4 in review round#2 was suggested when you were NOT using a selection model.] - Where are the results for first stage regression (in one of the many panels of Table 6?)? Where have they been discussed in detail in the manuscript? - The language is also quite confusing. I would suggest rewording this content for clarity.

We appreciated the reviewer’s recommendation to incorporate our two-stage analysis with a joint modeling approach and have operationalized this analysis within a generalized structural equation modeling (SEM) approach. This broader approach was selected as it allows for us to model our count and binary outcomes appropriately through the use of Poisson and logistic regression models, respectively. While the Heckman selection model is a special case of generalized SEM modeling, this modeling approach is limited in that it cannot accommodate non-binary outcomes and its application is limited to selection due to censoring/non-observation of data. We have also added further detail to the methods section and results section as requested by the reviewer.

4) Outcome choice in Table 6: First of all, this table in its current form in unreadable. The table doesn’t fit the page, different panels have been thrown together without appropriate labelling, the text description is unclear. What is the sample size for this analysis? Outcomes chosen by authors for Table 6: Number of ANC visits – This was observed at which time-window, the last month of pregnancy (as you mentioned in your response)? Please mention this in brackets as well in the results section so that clarity is maintained. Place of delivery – This choice is fine as it doesn’t depend on any particular choice of time window. Number of post-partum visits – Was this asked in the first month postpartum (as mentioned in line 311?)? Isn’t that shorter/different from the Indian guideline which is meant for 42 days rather than a month (line 330)?

The analytic sample for Table 6 analyses is limited to women who were 6 or more months postpartum at the time of interview, which is now the consistent sample for all data presented in the paper. Our outcomes are specified in the measures section of our manuscript. We have added the sample size to the top row of each column.

5) Similar to item #4. # Another item which is still unaddressed (was raised by Reviewer#1 in the last round and previously by me as well) is describing WHY results are different for the two states. Authors describe the state of ICDS in these two states and mention few other statistics but as Reviewer#2 mentioned this apparent difference has still not been rationalized for the reader.

We have edited this paragraph for further clarity on potential differences in the results between the two states.

The issues related to empirical analysis/writing/layout/lack of clarity remain in the manuscript. Particularly, the lack of clarity/convincing arguments is a recurring issue in the manuscript where the content doesn’t flow/connect across sections and thus creates confusion.

Minor Comments:

1) Reference # 5 & 52 are same, retain only one.

Edited

2) Line 250 mentions “Village matched” this creates confusion as the content of the companion paper is not a part of this draft. Either describe this approach in a footnote or choose alternate wording.

We have added a citation, so that the reader can get more information if desired. We felt it was important to provide some context on the larger study and design to help inform the interpretation and also be transparent about where this data comes from

3) Line 239-240: “We hypothesize that natal home migration will lead to a disruption in the continuum of care and therefore lower access and utilization of services (Figure 1).” Literature provided by the authors suggests a movement in either direction, how was this particular hypothesis (i.e. disruption) selected?

Thank you for this point, we have added “An alternate hypothesis, in the opposite direction, is that women could receive better care at their natal home.”

4) Line 284: Replace “12m” with less than 12 months age

Edited

5) Reference to Appendix items 1 & 3 missing in draft.

-Apologies. Based on reviewer feedback we have now integrated appendix 1 into table 1, and removed appendix 3 because we changed our models.

6) Table notes should be expanded, at this stage it is not possible to read the tables independent of the text. Describe the model used, dependent, key independent variable/s, mention what * represent i.e. what is the hypothesis being tested.

We have revised each of our tables carefully in order to improve the clarity of our presentation, expanding our titles and adding notes to each table so that the tables are able to be understood independent of the text.

7) Mention in text and table what is IQR and IRR (along with their full form).

Added

8) The legend description of mathematical equation is incorrect. It should be βi and not β1-i.

Thank you for identifying this error; we have corrected the model specification.

R2

Overall, I think this paper is interesting and does paper does contribute to the literature by documenting an under investigated phenomena. The paper does contribute a documentation of an under investigated phenomena which potentially has some profound implications for policy. The analysis, although limited in scope does appear to be a reasonable approach. The discussion and conclusion do follow from the results. However, the paper does not extend much beyond documenting this phenomenon. Given that the main contribution of the paper is to document the phenomena, it may be worthwhile emphasising why this is important to document and what implications these results have, especially for health outcomes. I can see that documenting this phenomenon is potentially important for policymakers and for future research. For researchers, not knowing that mothers migrate during pregnancy or soon thereafter, is likely causing survey sampling issues because they migrate. Also, the authors make clear that many policies designed to support mothers during pregnancy are not designed to accommodate maternal migration, and therefore is important to document. Overall, the authors have addressed reviewers’ comments. I am happy with the changes that were made, and the authors have improved their responses, compared to last time. However, there are still a handful of comments that need addressing prior to publication. These are small details but are necessary for publication.

Thank you so much for your ongoing feedback.

The authors state that three items were included in the appendix, however I see only 2 tables. Specifically, the item which was requested “Appendix 1: Sociodemographic Characteristics of women who migrated at all compared to women who did not migrate at any point” is not included and missing, although the caption is included. Authors need to include this table in the appendix.

-Apologies, we decided that this information was important and we moved it to Table 1 of the main manuscript.

In Appendix Table 3, it is unclear what is meant by “district-level variable” and I don’t understand what the coefficient on this variable means. It was district fixed effects which were requested; therefore, I would not expect “district” to be included in the table with a coefficient. The reviewers requested a district fixed effect regression, and therefore the reviewers need to estimate this model and include this in the appendix. Usually, a district- level fixed effect model would not include “district” as an additional variable with a coefficient in the table.

Please see our response to reviewer 1, comment 2. Subsequently, we have removed this appendix.

Table 6 is unreadable, as it spans outside of the range of the page, and therefore I couldn’t read it. Authors need to make sure that pages with large tables, such as these, are either landscape, or scaled so that they are readable.

We have revised the layout of this table.

This comment was not addressed: “Reviewer 2’s comment: “Tables 3, 4: Are the significance levels indicative of difference between states? Mention this detail in table footnotes.” In would usually prefer that tables be readable on their own, without the need for inspecting the text. In other words, full details of how the numbers in the table were calculated should be included in the footnotes. Putting *** p<0.” does not tell the reader what test was done to reach that result, was it the difference in means between states, or was it the test of coefficients being different from zero.”

Changes were made to other tables, but not to Tables 3 and 4. Again, what do these stars denote? Is this the significance in a t-tests of differences in means? Whatever the test used, include in footnote exactly what test was done to get these p-values. Be precise about where these p-values come from. The authors need to include precise detail of what test of differences was done, and state that they are testing the difference between states, in the footnote of Tables 3 and 4.

-Apologies, we have added to our notes under each table that these represent the results of tests of differences in the distribution of these factors between the states using chi-squared tests. As noted in our responses to reviewer 1, we have also revised the titles and added further text below the tables to improve clarity.
